# AHR Deficiency Exacerbates Hepatic Cholesterol Accumulation via Inhibiting Bile Acid Synthesis in MAFLD Rats

**DOI:** 10.3390/ijms27010349

**Published:** 2025-12-29

**Authors:** Junjiu Xu, Pengwei Liu, Yuling Wu, Hongxiu He, Dandan Hu, Jianhua Sun, Jing Chen, Ying Tian, Likun Gong

**Affiliations:** 1 School of Chinese Materia Medica, Nanjing University of Chinese Medicine, Nanjing 210046, China; jjxu@cdser.simm.ac.cn (J.X.); wuyuling945@zidd.ac.cn (Y.W.); hongxiu.he@lglab.ac.cn (H.H.); 2 Center for Drug Safety Evaluation and Research, State Key Laboratory of Drug Research, Shanghai Institute of Materia Medica, Chinese Academy of Sciences, 501 Haike Road, Shanghai 201203, China; sunjianhua319@zidd.ac.cn (J.S.); jingchen@simm.ac.cn (J.C.); 3 State Key Laboratory of Discovery and Utilization of Functional Components in Traditional Chinese Medicine & School of Pharmaceutical Sciences, Guizhou Medical University, Guizhou 561113, China; liupengwei507@zidd.ac.cn; 4 Zhongshan Institute for Drug Discovery, Shanghai Institute of Materia Medica, Chinese Academy of Sciences, Zhongshan 528400, China; hudandan@zidd.ac.cn; 5 China-Serbia “Belt and Road” Joint Laboratory for Natural Products and Drug Discovery, Shanghai Institute of Materia Medica, Chinese Academy of Sciences, 555 Zu-Chong-Zhi Road, Zhangjiang Hitech Park, Shanghai 201203, China

**Keywords:** MAFLD, AHR, bile acid, cholesterol, gut microbiota

## Abstract

Metabolic-dysfunction-associated fatty liver disease (MAFLD) is a chronic liver disease characterized by abnormal lipid metabolism. The aryl hydrocarbon receptor (AHR) is a ligand-dependent transcription factor involved in regulating multiple physiological processes. Recent studies have demonstrated that AHR exerts a multifaceted regulatory role in liver diseases by integrating metabolic and immune signaling pathways; however, the specific role of AHR in MAFLD is not clear. In our work, a rat model of MAFLD was established by feeding wild-type (WT) and AHR knockout (AHR^−/−^) rats with a high-fat, high-fructose, and high-cholesterol diet (HFHFrHCD) for 10 weeks, and then the liver injury markers, lipid-related biochemical indices and liver histopathology were examined to elucidate the effect of AHR on MAFLD progression. We discovered that AHR deficiency can elevate plasma transaminase levels, increase hepatic triglyceride (TG) and total cholesterol (TC), and exacerbate insulin resistance (IR) under an overnutrition environment. Subsequently, liver transcriptome and RT-qPCR were performed to investigate the underlying mechanism, which revealed that the hepatic bile acid synthesis was inhibited because of lower *Cytochrome P450 Family 7 Subfamily A Member 1 (CYP7A1)* expression in the liver when AHR was knockout. Additionally, intestinal flora dysbiosis occurred in AHR^−/−^ rats fed with HFHFrHCD, which might also contribute to the hepatic cholesterol accumulation. Taken together, our results suggested that AHR might play an important role in regulating cholesterol metabolism by inhibiting bile acid synthesis and breaking the steady state of the gut microbiota during the MAFLD progression.

## 1. Introduction

Nonalcoholic fatty liver disease (NAFLD) is a chronic liver disease characterized by excessive hepatic fat accumulation and metabolic dysfunction [1], which in severe cases can progress to hepatic fibrosis, cirrhosis, and hepatocellular carcinoma. However, due to the heterogeneity of NAFLD and its inability to reflect current understanding of the disease pathogenesis, an international panel of experts unanimously recommended renaming this disease as metabolic-dysfunction-associated fatty liver disease (MAFLD) [2]. This renaming emphasizes the pathogenic interaction between fatty liver and systemic metabolic abnormalities and applies more inclusive diagnostic criteria. Notably, a series of studies have demonstrated that compared with the traditional NAFLD diagnostic criteria, the MAFLD criteria are more aligned with clinical needs and more effective in identifying populations at high risk of adverse liver outcomes [3,4,5,6].

Clinically, MAFLD affects approximately one-third of the global population, with a significantly higher prevalence in overweight and obese individuals [7]. As the global prevalence and severity of obesity continue to rise, the public health burden of MAFLD is expected to increase. In MAFLD, the dysregulated lipid metabolism is a key driver of disease progression. Hepatic lipid homeostasis is coordinated by three core pathways: fatty acid uptake and export, de novo lipogenesis (DNL), and fatty acid β-oxidation. Disruption of the balance among these pathways results in excessive hepatic lipid accumulation, which further exacerbates disease progression [8]. In addition to lipid metabolic turbulence, accumulating evidence has demonstrated that cholesterol metabolism dysregulation also plays a pivotal role in MAFLD pathogenesis. Physiologically, cholesterol homeostasis is maintained by intracellular cholesterol-sensing mechanisms and transcription factors, which collectively regulate cholesterol synthesis, uptake, and excretion. Impairment of these regulatory pathways disrupts the balance of cholesterol metabolism, ultimately leading to cholesterol toxicity and hepatic lipid accumulation [9].

Studies have shown that transcription factors play a key role in the pathogenesis and progression of MAFLD by regulating multiple metabolic pathways, particularly lipid metabolism [10,11]. Aryl hydrocarbon receptor (AHR), a ligand-activated transcription factor (activated by both endogenous and exogenous ligands), is highly expressed in the liver. Classically, AHR is known for regulating xenobiotic metabolic genes; it is also recognized as a key regulator of immune regulation, cell proliferation, and differentiation that underlies a broad spectrum of liver diseases [12]. Increasing evidence highlights the AHR’s role in regulating lipid metabolism and the progression of obesity, NAFLD, and type 2 diabetes (T2D) [13,14]. Preclinical studies have demonstrated that modulation of AHR signaling can significantly alter these disease phenotypes. Moving beyond its canonical function in xenobiotic metabolism, recent findings reveal that AHR activation impairs hepatic insulin signaling, thereby contributing to systemic insulin resistance and glucose intolerance [15]. This effect is facilitated by the enhanced hepatic uptake of exosomal phosphatidylcholine. Furthermore, AHR regulates mitochondrial homeostasis by modulating BNIP3-dependent mitophagy [14,16]. The deficiency in AHR leads to mitochondrial dysfunction and increased reactive oxygen species (ROS), which exacerbates hepatocellular injury [17]. Notably, AHR has emerged as a pivotal mediator of the gut–liver axis. Microbiota-derived tryptophan metabolites, such as indoles, activate AHR in both the intestine and liver [18]. Concurrently, AHR activation promotes intestinal barrier integrity by upregulating the tight junction proteins, including Zonula Occludens-1 (ZO-1) and occludin [19]. Conversely, the AHR deficiency increases gut permeability, facilitating the translocation of microbial products such as lipopolysaccharide (LPS) into the portal circulation. This process triggers chronic low-grade inflammation and accelerates the development of metabolic syndrome. Collectively, these findings position AHR as a central regulator that integrates environmental and microbial signals to control metabolic homeostasis, highlighting its potential as a promising therapeutic target for hepatic and metabolic disorders [20].

To facilitate the investigation of MAFLD/NAFLD pathogenesis and drug development, researchers have established various MAFLD animal models, primarily by modifying dietary compositions. Notably, a typical Western diet—characterized by high fat, high fructose, and high cholesterol—has been widely used for MAFLD modeling. Compared with other approaches, this dietary regimen better recapitulates the clinical metabolic background of human MAFLD, as it not only induces steatohepatitis but also elicits obesity and insulin resistance (key features of metabolic syndrome) [21,22]. However, none of the existing studies on the AHR in MAFLD have employed this clinically relevant dietary model. Given that AHR is well-documented to regulate lipid metabolism and that the global incidence of MAFLD continues to rise, it remains unknown whether AHR exerts consistent regulatory effects across different MAFLD models. Therefore, the present study aimed to elucidate the role of AHR in MAFLD progression by establishing a chronic MAFLD animal model fed HFHFrHCD.

## 2. Results

### 2.1. AHR Knockout Exacerbates HFHFrHCD-Induced Liver Injury and Insulin Resistance

**Figure 1:**
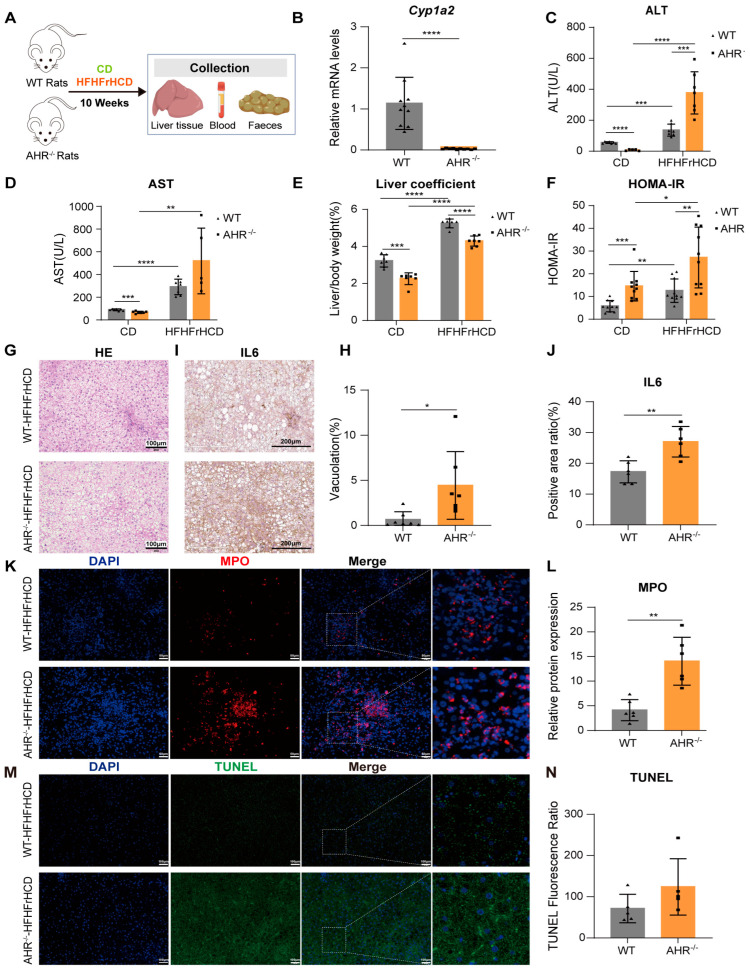
AHR deficiency exacerbates HFHFrHCD-induced liver injury, inflammation, and insulin resistance. (**A**) Schematic diagram of the animal experimental design. (**B**) Hepatic *Cyp1a2* mRNA expression levels in WT and AHR knockout rats. (**C**–**F**) Plasma ALT (**C**), AST levels (**D**), liver coefficients (**E**), and insulin resistance (**F**) in WT and AHR knockout rats after 10 weeks of CD or HFHFrHCD feeding. (**G**,**H**) Representative HE staining images of liver (**G**) and quantitative analysis of vacuolation area (**H**) in WT and AHR knockout rats after HFHFrHCD feeding. (**I**,**J**) Representative immunohistochemical staining images (**I**) and quantitative analysis (**J**) of hepatic IL-6 in WT and AHR rats after HFHFrHCD feeding. (**K**,**L**) Representative immunofluorescence staining images of hepatic MPO (**K**), and quantification analysis of MPO fluorescence intensity (**L**) in WT and AHR rats after HFHFrHCD feeding. (**M**,**N**) Representative TUNEL staining images (**M**) and quantification of TUNEL-positive cells (**N**) in the liver. Data are shown as mean ± SD, statistical significance: * *p* < 0.05, ** *p* < 0.01, *** *p* < 0.001, **** *p* < 0.0001.

To investigate the role of AHR in MAFLD progression, WT and AHR knockout rats were fed HFHFrHCD and a control diet (CD) for 10 weeks (Figure 1A). Body weight change and food intake were shown in Figure S1. Expression of *Cytochrome P450 Family 1 Subfamily A Member 2 (CYP1A2)*, a downstream gene of AHR, was significantly reduced, indicating that AHR was completely knocked out in the rat liver (Figure 1B). As shown in Figure 1, the lack of AHR aggravated HFHFrHCD-induced elevation of plasma transaminase levels (Figure 1C,D) and degree of insulin resistance (Figure 1F) suggest that AHR deficiency promotes HFHFrHCD-induced liver injury. HFHFrHCD led to a significant increase in the liver coefficients of rats. In contrast, AHR-knockout rats on the same diet exhibited a marked reduction in this parameter. (Figure 1E). HE staining of the liver showed larger vacuolar structures in the AHR^−/−^-HFHFrHCD group (Figure 1G,H), which indicated that the more severe lipid accumulation had occurred. To further evaluate the effect of AHR on HFHFrHCD-induced liver injury, we examined the levels of inflammatory markers IL-6, myeloperoxidase (MPO) and apoptosis in the liver tissues. The results showed that the absence of AHR significantly increased the levels of IL-6 and MPO in the HFHFrHCD group (Figure 1I–L), while the apoptosis levels tended to be elevated without significant differences (Figure 1M,N). The above results suggest that AHR knockout can accelerate HFHFrHCD-induced hepatic damage.

### 2.2. AHR Knockout Facilitates HFHFrHCD-Induced Lipid Accumulation

**Figure 2:**
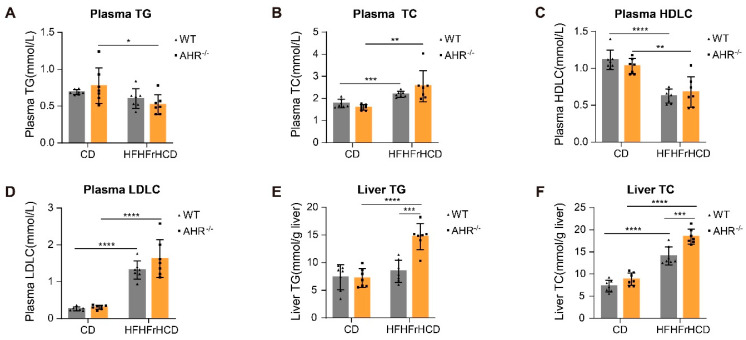
AHR deficiency exacerbates HFHFrHCD-induced hepatic lipid accumulation. (**A**–**D**) Plasma TG (**A**), TC (**B**), HDLC (**C**), and LDLC (**D**) levels in WT and AHR^−/−^ rats after 10 weeks of CD or HFHFrHCD feeding. (**E**,**F**) Liver TG (**E**) and TC (**F**) levels in WT and AHR^−/−^ rats after 10 weeks of CD or HFHFrHCD feeding. Data are shown as mean ± SD, statistical significance: * *p* < 0.05, ** *p* < 0.01, *** *p* < 0.001, **** *p* < 0.0001.

Since AHR deficiency resulted in more pronounced vacuolization in the livers of rats in the HFHFrHCD groups, we speculated that AHR might be associated with lipid homeostasis, allowing us to examine lipid levels in the plasma and liver. The analysis of plasma lipids revealed that HFHFrHCD feeding significantly increased Total Cholesterol (TC) (Figure 2B) and low-density lipoprotein cholesterol (LDLC) (Figure 2D) levels and decreased high-density lipoprotein cholesterol (HDLC) levels (Figure 2C) in both WT and AHR knockout rats compared to the CD group. However, HFHFrHCD feeding significantly reduced plasma Triglyceride (TG) levels in AHR knockout rats, with no significant effect in WT rats (Figure 2A). Moreover, the absence of AHR did not significantly aggravate plasma TC, LDLC, or HDLC levels (Figure 2B–D) affected by HFHFrHCD. In terms of hepatic lipids, HFHFrHCD induced hepatic TC accumulation with no effect on TG in WT rats, while HFHFrHCD-induced increases in TG and TC levels were more evident after AHR knockout (Figure 2E,F). This evidence indicates that the AHR knockout aggravates HFHFrHCD-induced hepatic lipid accumulation without affecting blood lipid levels.

### 2.3. Expression of Lipid Metabolism-Related Genes Dysregulated in AHR Knockout Rats Under the Pressure of HFHFrHCD

**Figure 3:**
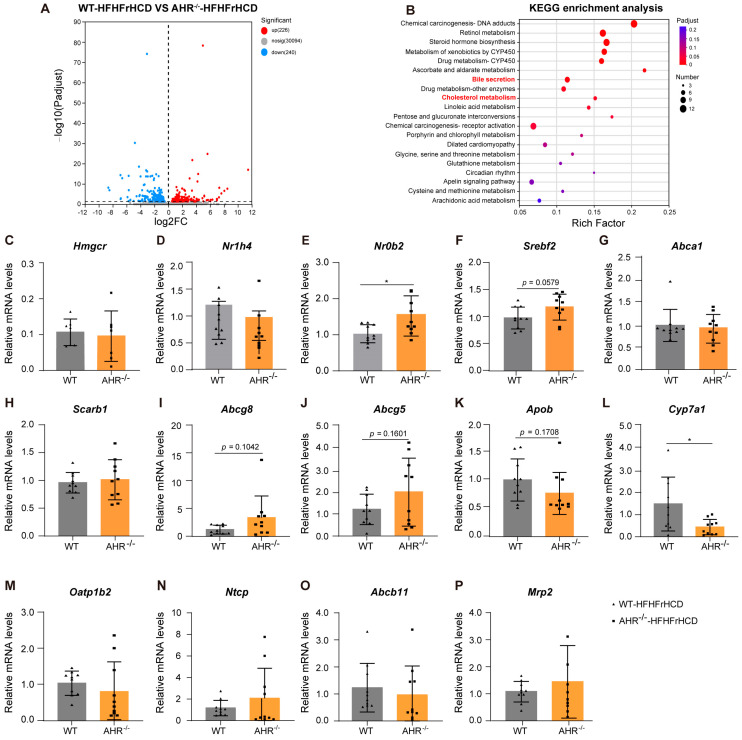
AHR deficiency exacerbates HFHFrHCD-induced abnormalities in the expression of lipid metabolism-related genes. (**A**) Volcano plot of DEGs in liver tissues between the WT-HFHFrHCD and AHR^−/−^-HFHFrHCD groups. (**B**) KEGG enrichment analysis of DEGs in liver tissues between the WT-HFHFrHCD and AHR^−/−^-HFHFrHCD groups. (**C**–**K**) Hepatic mRNA levels of genes related to cholesterol synthesis (**C**), metabolism-related transcription factors (**D**–**F**), transport (**G**,**H**) and excretion (**I**–**K**) in WT and AHR^−/−^ rats fed a HFHFrHCD. (**L**–**P**) Hepatic mRNA levels related to bile acids synthesis (**L**), bile acid/cholesterol metabolism-related transcription factors (**D**,**E**), intake (**M**,**N**), and excretion (**O**,**P**) in WT and AHR^−/−^ rats fed a HFHFrHCD. Data are shown as mean ± SD, statistical significance: * *p* < 0.05.

To further explain how AHR deletion promotes HFHFrHCD-induced abnormalities in lipid metabolism, RNA sequencing (RNA-seq) analysis was performed on the WT-HFHFrHCD and AHR^−/−^-HFHFrHCD groups. Differentially expressed genes (DEGs) were identified using thresholds of fold change > 1.5 and *p*-value < 0.05 for the comparison between the WT-HFHFrHCD group and the AHR^−/−^-HFHFrHCD group. Under HFHFrHCD conditions, AHR deficiency mainly affected the expression of 466 downstream genes, out of which 226 were upregulated, and a total of 240 were downregulated (Figure 3A). The pathways involved in these DEGs included chemical carcinogenesis, retinol metabolism, steroid hormone biosynthesis, metabolism of xenobiotics by CYP450, drug metabolism, ascorbate and aldarate metabolism, bile secretion, cholesterol metabolism, and so on (Figure 3B). Of these, the most relevant to our disease model are bile secretion and cholesterol metabolism. Bile facilitates fat digestion, with bile acids being its core components. Based on this, we propose that AHR knockout primarily induces hepatic lipid accumulation by regulating bile acid metabolism and cholesterol metabolism.

Next, we examined the expression levels of key genes involved in bile acid metabolism and cholesterol metabolism, including transcription factors related to bile acid/cholesterol metabolism (*Nr1h4* (FXR), *Nr0b2* (SHP), *Srebf2* (SREBP2)) (Figure 3D–F), cholesterol synthesis (*Hmgcr*) (Figure 3C), cholesterol transport (*Abca1*, *Scarb1*) (Figure 3G,H), cholesterol excretion (*Abcg8*, *Abcg5*, *Apob*) (Figure 3I–K), as well as bile acid synthesis(*Cyp7a1*) (Figure 3L), uptake (*Oatp1b2*, *Ntcp*) (Figure 3M,N), and excretion (*Abcb11*, *Mrp2*) (Figure 3O,P). Except for *Nr0b2*(SHP) and *Cyp7a1*, AHR deficiency did not significantly alter the expression of these genes. To distinguish the effects of overnutrition from those of AHR deficiency, differential gene expression was also analyzed between the WT-CD and AHR^−/−^-CD groups. Under control diet conditions, the pathways associated with these DEGs were comparable to those observed under overnutrition conditions, except for cholesterol metabolism (Figure S2A). Moreover, the marked suppression of *Cyp7a1* was also observed in the AHR^−/−^-CD groups (Figure S2B). Since HFHFrHCD could increase hepatic TG content in the AHR knockout group, we also presented the genes related to lipid uptake and lipoproteins, key regulators of Very Low-Density Lipoprotein (VLDL) assembly and secretion, and *De Novo* lipogenesis in the RNA-sequencing data (Figure S3), where an increasing trend in the genes of *De Novo* lipogenesis was observed, but without a statistically significant difference.

These results illustrate that AHR deficiency may cause abnormal hepatic lipid accumulation, primarily by suppressing bile acid synthesis, in MAFLD.

### 2.4. Effect of AHR Deficiency on Hepatic and Fecal Bile Acid Profiles in MAFLD Rats

Given that bile acid dysregulation is closely associated with dyslipidemia and insulin resistance in MAFLD, and that we discovered that AHR deficiency may suppress bile acid synthesis, we performed comparative analysis of hepatic and fecal bile acid profiles between the WT-HFHFrHCD and AHR^−/−^-HFHFrHCD groups to investigate the effect of AHR knockout on hepatic and fecal bile acids.

**Figure 4:**
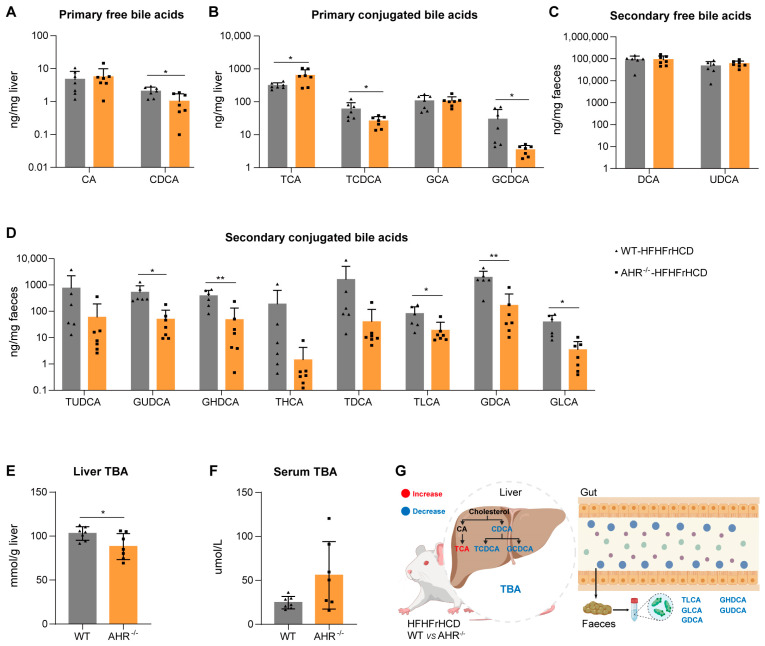
AHR deficiency exacerbates HFHFrHCD-induced disorders of bile acid metabolism. (**A**,**B**) Hepatic levels of primary free (**A**) and conjugated (**B**) bile acids in WT-HFHFrHCD and AHR^−^/^−^-HFHFrHCD groups. (**C**,**D**) Fecal levels of secondary free (**C**) and conjugated (**D**) bile acids in the WT-HFHFrHCD and AHR^−/−^-HFHFrHCD groups. (**E**,**F**) Hepatic (**E**) and plasma (**F**) levels of total bile acids in WT-HFHFrHCD and AHR^−/−^-HFHFrHCD groups. (**G**) Graphic summary. Data are shown as mean ± SD, statistical significance: * *p* < 0.05, ** *p* < 0.01.

Under conditions of overnutrition, AHR deletion significantly decreased hepatic levels of the primary free bile acid Chenodeoxycholic Acid (CDCA) (Figure 4A) and the primary conjugated bile acids Taurochenodeoxycholic Acid (TCDCA) and Glycochenodeoxycholic Acid (GCDCA) (Figure 4B). It also reduced fecal levels of the secondary conjugated bile acids Glycoursodeoxycholic Acid (GUDCA), Glycohyodeoxycholic Acid (GHDCA), Taurolithocholic Acid (TLCA), Glycodeoxycholic Acid (GDCA) and Glycolithocholic Acid (GLCA) (Figure 4D). No significant effect of AHR deficiency was observed on the levels of fecal secondary free bile acids, such as Lithocholic Acid (LCA), Deoxycholic Acid (DCA) and Ursodeoxycholic Acid (UDCA) (Figure 4C). Conversely, the hepatic primary conjugated bile acid Taurocholic Acid (TCA) was significantly increased (Figure 4B), a change that was opposed to the trends observed for other bile acids. These results indicated that AHR deletion significantly altered both hepatic and fecal BA compositions in HFHFrHCD-fed rats.

To investigate the effect of AHR on total bile acids (TBA) levels under HFHFrHC conditions, we measured hepatic and plasma TBA concentrations. The results showed that AHR deficiency demonstrated a significant reduction in hepatic TBA levels (Figure 4E). In contrast, plasma TBA levels showed a non-significant increasing trend (Figure 4F). AHR knockout did not significantly affect hepatic bile acid uptake or excretion, as the expression of key transporters remained unchanged (Figure 3K–N). A plausible explanation for these observations is that reduced bile acid synthesis in AHR-deficient hepatocytes directly contributes to decreased hepatic TBA levels (Figure 4G).

### 2.5. Effect of AHR Deficiency on Gut Microbiota Diversity and Composition in MAFLD Rats

**Figure 5:**
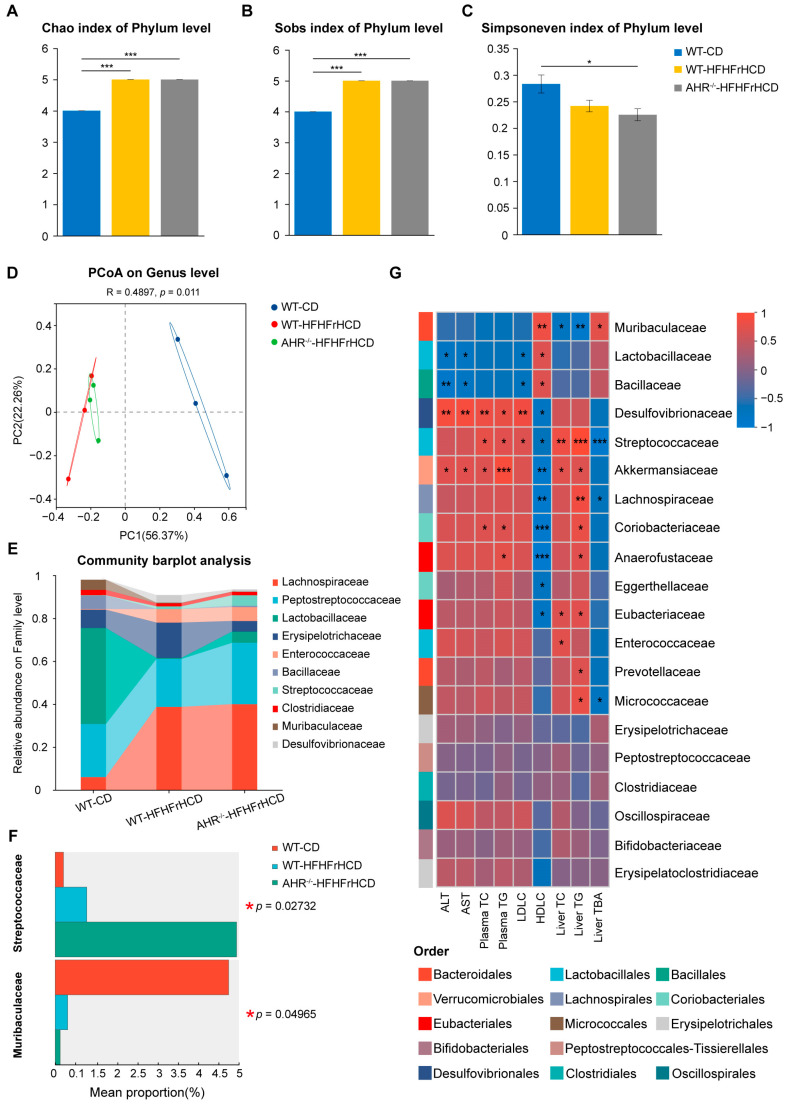
AHR deficiency exacerbates HFHFrHCD-induced gut flora disruption. (**A**–**C**) Alpha diversity index of gut microbiota at the phylum level: (**A**) Chao, (**B**) Sobs, and (**C**) Simpson’s index. (**D**) PCoA analysis of gut microbiota at the genus level in the WT-CD, WT-HFHFrHCD, and AHR^−/−^-HFHFrHCD groups. (**E**) The community bar chart displays the top 10 families. (**F**) Differential species at the family level. (**G**) Heatmap of correlation. Data are shown as mean ± SD, statistical significance: * *p* < 0.05, ** *p* < 0.01, *** *p* < 0.001.

Accumulating evidence shows that alterations in gut microbial diversity and composition are associated with the onset and progression of MAFLD. To elucidate the specific role of AHR in this context, we performed microbial diversity analysis on fecal samples from WT-CD, AHR^−/−^-CD, WT-HFHFrHCD, and AHR^−/−^-HFHFrHCD groups. Gut microbiota analysis revealed that AHR knockout did not alter the gut microbiota diversity in rats on a normal diet (Figure S4A–C). Further gut microbiota analysis demonstrated that both WT-HFHFrHCD and AHR^−/−^-HFHFrHCD groups exhibited elevated Chao and Sobs indices, while the AHR^−/−^-HFHFrHCD group displayed a reduced Simpson’s index compared to the WT-CD group (Figure 5A–C). Furthermore, a significant difference in β-diversity was observed among the groups, as evidenced by the PCoA analysis (*p* = 0.011) (Figure 5D).

At the family level, HFHFrHCD feeding increased Streptococcaceae and decreased Muribaculaceae abundance compared to the CD group. Notably, AHR knockout exacerbated these HFHFrHCD-induced changes, resulting in a further increase in Streptococcaceae and a further decrease in Muribaculaceae in AHR^−/−^-HFHFrHCD rats relative to their WT-HFHFrHCD counterparts (Figure 5E,F).

To elucidate the relationship between liver dysfunction and gut microbiota alterations, we performed a correlation analysis. Correlation analysis revealed significant associations between the differential flora and key indices. Specifically, Muribaculaceae abundance was negatively correlated with hepatic TC and TG levels but positively correlated with hepatic TBA levels. The abundance of Streptococcaceae was positively correlated with hepatic TC and TG, but negatively correlated with hepatic TBA levels (Figure 5G). These findings demonstrate that AHR deficiency induced further alterations in the gut microbial community structure of MAFLD rats, which were closely associated with the aggravation of hepatic fat accumulation.

## 3. Discussion

MAFLD is a highly prevalent chronic liver condition marked by hepatic steatosis and metabolic dysregulation [23]. Numerous studies have demonstrated a strong association between the gut-liver axis and MAFLD. The gut-liver axis refers to the bidirectional interaction between the intestine and the liver. Intestinal abnormalities, including gut microbiota dysbiosis and intestinal barrier impairment, can trigger hepatic dysfunction via the gut-liver axis. Given that the hepatic portal vein, which originates from the intestine, supplies 75% of the liver’s blood flow, the liver is the first organ to be affected [24]. Conversely, the liver affects the gut microbiota by influencing bile secretion.

AHR, a ligand-activated transcription factor highly expressed in the intestine and liver, is a crucial regulator of intestinal barrier homeostasis [25]. Impairment of the gut barrier allows intestinal metabolites, bacteria, and endotoxins to translocate into the enterohepatic circulation, adversely affecting the liver. Consequently, AHR represents a key player in the gut-liver axis, positioning it as a factor potentially relevant to MAFLD pathogenesis.

Previous studies investigating the role of AHR in the progression of MAFLD have predominantly employed high-fat diet-induced models [26], which neglect the impact of dietary composition and thus fail to accurately simulate the clinical context of MAFLD. Considering that different dietary patterns induce phenotypic variations in the disease, and to align with the clinical features of MAFLD, we established an MAFLD model using WT and AHR knockout rats fed a high-fat, high-fructose, high-cholesterol diet. Interestingly, rather than alleviating the key features of MAFLD, AHR deletion exacerbated the progression of MAFLD. Specifically, compared with WT rats subjected to HFHFrHCD, AHR deficiency aggravated HFHFrHCD-induced liver injury, insulin resistance, and hepatic lipid accumulation (particularly triglycerides and cholesterol). These findings contradict those of previous studies, suggesting that dietary differences exert a significant influence: the previous models do not reflect the dietary profiles of populations at risk for MAFLD, and the actual role of AHR in MAFLD may be diametrically opposed to prior assumptions. Notably, AHR knockout rats exhibited reduced liver coefficients regardless of diet, which is likely attributable to the known role of AHR in liver development: its absence impairs hepatocyte proliferation, leading to reduced liver size and hepatocyte dimensions [27]. This may mask the underlying steatosis-associated hepatomegaly.

Our findings indicated that AHR knockout under HFHFrHCD conditions led to hepatic TG accumulation. From the heatmap analysis of DEGs, we discovered there was an increasing trend in the genes responsible for de novo lipogenesis (DNL). Moreover, we also observed a decreasing trend in hepatic *Apob* expression in AHR-deficient rats. As ApoB serves as the essential structural scaffold of very-low-density lipoprotein (VLDL) particles, its decrease would be predicted to impair VLDL assembly, for which secretion is the major route for hepatic TG efflux [28]. Therefore, we inferred that the observed phenotype reduced TG release into the circulation, and the intrahepatic TG retention might be the comprehensive effect of these genes.

Our transcriptome analyses revealed that HFHFrHCD triggers aberrant gene expression when AHR is knocked out, among which bile secretion and cholesterol metabolism are strongly associated with MAFLD progression. Bile is a complex fluid synthesized by hepatocytes and stored in the gallbladder, while bile acids represent the most abundant organic component of bile. Critically, bile acids are indispensable for mediating bile’s core physiological functions, including emulsifying dietary fats to facilitate absorption and promoting cholesterol excretion processes that are central to the pathogenesis of MAFLD, as dysregulation of these pathways contributes to hepatic lipid accumulation and inflammation. Bile acids exist in either free or conjugated forms, with the latter formed through conjugation to glycine or taurine. Beyond their classical role in dietary lipid emulsification, bile acids are now recognized as key metabolic signaling molecules that regulate energy and lipid homeostasis [29]. Hepatic bile acid synthesis occurs predominantly via two pathways [30]: the classical (neutral) pathway, initiated by cholesterol 7α-hydroxylase (CYP7A1), and the alternative (acidic) pathway, mediated by sterol 27-hydroxylase (CYP27A1). The classical pathway accounts for approximately 75% of total bile acid production and represents the major route for cholesterol catabolism. In this pathway, CYP7A1 catalyzes the rate-limiting 7α-hydroxylation of cholesterol to yield 7α-hydroxy cholesterol. This intermediate is subsequently processed through a series of oxidation and reduction reactions, with sidechain 12α-hydroxylation catalyzed by CYP8B1, ultimately leading to the formation of cholic acid (CA). In contrast, the alternative pathway begins with CYP27A1-mediated hydroxylation at the C-27 position of cholesterol, yielding 27-hydroxycholesterol. This intermediate undergoes further 7α-hydroxylation by oxysterol 7α-hydroxylase (CYP7B1), ultimately producing chenodeoxycholic acid (CDCA). In the liver, CA and CDCA are conjugated to glycine or taurine to form primary conjugated bile acids—including taurocholic acid (TCA), glycocholic acid (GCA), taurochenodeoxycholic acid (TCDCA), and glycochenodeoxycholic acid (GCDCA)—thereby enhancing their water solubility and facilitating biliary secretion into the intestinal lumen. Within the intestine, gut microbiota mediated the biotransformation of these primary bile acids into secondary free bile acids, such as deoxycholic acid (DCA), lithocholic acid (LCA), and ursodeoxycholic acid (UDCA). A portion of these secondary bile acids is reabsorbed via enterohepatic circulation, while the remainder is further modified into secondary conjugated BAs (e.g., to form GDCA, TDCA, and GLCA) before fecal excretion. Cholesterol serves as the main precursor for primary bile acid synthesis, while this pathway represents a major route for eliminating excess cholesterol in humans. Consequently, we examined the expression levels of key genes involved in bile acid and cholesterol metabolism. Our findings revealed that AHR deficiency significantly suppressed hepatic bile acid synthesis (Figure S2B). However, we did not present the BA profile in WT and AHR-KO rats fed with a regular diet, which needs more experiments to confirm our conclusion.

To investigate the mechanism underlying the suppression of CYP7A1 expression by AHR knockout, we analyzed the expression of genes involved in upstream regulatory pathways. The hepatic FXR–SHP axis constitutes the core negative feedback loop controlling bile acid synthesis. The elevated intrahepatic bile acid levels activate FXR, leading to the induction of SHP, which in turn represses CYP7A1 transcription and thereby reduces bile acid production to restore metabolic homeostasis. Notably, following AHR deletion, FXR expression remained unchanged, while SHP expression was significantly upregulated (Figure 3E). This suggests the involvement of an alternative pathway that enhances SHP expression independently of increased FXR abundance. In this context, AHR appears to function as an inhibitor of SHP activation. Thus, AHR deficiency leads to elevated expression of SHP, resulting in exaggerated suppression of CYP7A1 and impaired bile acid synthesis, ultimately promoting hepatic cholesterol accumulation. Although AHR deficiency appeared to promote cholesterol synthesis (*Srebf2*), this effect was not statistically significant, possibly due to individual animal variations. This evidence clearly indicates that AHR knockout exacerbates HFHFrHCD-induced hepatic cholesterol accumulation by suppressing bile acid synthesis. Concurrently, in our rat experiments, decreased primary bile acid excretion was observed in the AHR^−/−^-HFHFrHCD groups. AHR deficiency suppresses hepatic bile acid synthesis, thereby impairing the conversion of cholesterol into bile acids and disrupting systemic cholesterol homeostasis. The resulting imbalance in the hepatic cholesterol-to-bile acid ratio reduces the capacity of bile to solubilize cholesterol within mixed micelles, promoting cholesterol supersaturation, nucleation, and crystal formation. Extrapolating to humans, insufficient primary bile acid synthesis, similar to that in AHR-deficient rats, may prevent the formation of cholesterol—solubilizing mixed micelles in the gallbladder. This could lead to cholesterol precipitation and stone formation, indicating that factors reducing AHR—mediated bile acid synthesis in humans might increase the risk of cholesterol gallstone disease. Such metabolic disturbances not only contribute to hepatocellular cholesterol accumulation but also increase susceptibility to cholesterol gallstone disease. BAs serve as a critical metabolic hub integrating gut-liver crosstalk and play a vital role in lipid digestion and absorption. Given the liver’s central role in the BA synthesis and metabolism, we comprehensively analyzed the hepatic and fecal BA profiles.

Our findings demonstrate that AHR deficiency suppresses hepatic BA synthesis by downregulating CYP7A1 expression, leading to a marked reduction in the levels of most BAs in both the liver and faeces. Interestingly, AHR deficiency specifically elevated hepatic TCA levels in rats fed HFHFrHCD. This indicates that AHR loss not only diminishes the total hepatic BA pool but also alters its composition. The underlying mechanism driving this specific TCA accumulation warrants further investigation. As the BA synthesis represents the dominant pathway for cholesterol catabolism and elimination, its suppression—concurrent with enhanced cholesterol synthesis—promotes the pathological accumulation of hepatic cholesterol, thereby potentiating liver injury. This mechanism likely constitutes a key pathway through which AHR knockout exacerbates hepatic steatosis and damage. Furthermore, the observed alterations in BA profiles, particularly the reduction in secondary BAs, may disrupt gut microbial homeostasis, as BAs are known to exert antimicrobial effects and shape the intestinal microbiota composition.

The balance of the gut microbiota is critical for maintaining homeostasis of the gut-liver axis, and microbiota imbalance is strongly associated with liver disease [31]. Our microbial diversity analysis revealed that AHR deficiency does not alter gut microbial composition under standard dietary conditions (Figure S4). However, it exacerbated HFHFrHCD–induced gut dysbiosis and significantly altered the gut microbial composition. These changes may arise from AHR-dependent regulation of intestinal mucus production and antimicrobial protein expression, suggesting a protective role for AHR in preserving microbial balance during metabolic stress. Specifically, we observed a marked reduction in the abundance of Muribaculaceae and a concurrent increase in Streptococcaceae in the intestines of AHR^−/−^ rats fed a HFHFrHCD. Most studies illustrate that an elevated abundance of Streptococcaceae is well-documented in the gut of NAFLD patients, while a reduction of the mucus-associated probiotic family Muribaculaceae was observed in NAFLD mouse models [32,33,34,35,36,37,38,39,40,41]. Furthermore, Muribaculaceae abundance was negatively correlated with hepatic triglyceride (TG), total cholesterol (TC), and HOMA-IR levels. Our findings align with previous reports and confirm that AHR knockout aggravates the gut dysbiosis associated with metabolic dysfunction.

Taken together, the present study is the first to utilize AHR-knockout rats to establish a model of HFHFrHCD-induced MAFLD and to investigate the role of AHR in its pathogenesis. Contrary to what might be expected, we found that AHR deletion did not ameliorate but rather exacerbated MAFLD progression. This is likely due to the inhibition of bile acid synthesis, specifically through the downregulation of CYP7A1, a key enzyme in bile acid production, which reduces the conversion of cholesterol to bile acids. Additionally, AHR deficiency altered the gut microbiota abundance in MAFLD rats, characterized by a decrease in Muribaculaceae and an increase in Streptococcaceae. This disruption of the gut microbiota’s steady state also contributes to the progression of MAFLD. Collectively, these effects synergistically promoted hepatic lipid accumulation and injury.

## 4. Materials and Methods

### 4.1. Animal Experiments

All experiments were conducted using male Sprague-Dawley (SD) rats (*n* = 20) purchased from the Laboratory Animal Center of the Shanghai Institute of Planning, Parenthood Research (Shanghai, China). AHR^−/−^ rats (*n* = 20) were obtained from the National Resource Center for Mutant Rats, Institute of Laboratory Animal Sciences (ILAS), Chinese Academy of Medical Sciences (Beijing, China), and maintained at the animal facility of the Shanghai Institute of Materia Medica, Chinese Academy of Sciences (Shanghai, China). Before group assignment, animals were weight-matched and then randomly allocated to experimental groups to minimize bias due to body weight differences. Rats were housed under specific pathogen-free (SPF) conditions with a 12-h light/dark cycle and had ad libitum access to water and a standard chow diet unless otherwise specified. The control group received the standard chow diet, while model groups were fed a customized high-fat, high-fructose, high-cholesterol diet (HFHFrHCD; 40% kcal from fat [primarily palm oil], 20% kcal from fructose, and 2% *w*/*w* cholesterol) for 10 weeks (*n* = 10 per diet per genotype). After an overnight fast (8–10 h), animals were anesthetized, and terminal blood and tissue samples were collected for downstream analyses. All procedures involving animals were approved by the Institutional Animal Care and Use Committee (IACUC) of the Shanghai Institute of Materia Medica, Chinese Academy of Sciences, and carried out in strict accordance with institutional and national guidelines for the care and use of laboratory animals (IACUC: 2022-05-GLK-26).

### 4.2. Biochemical Assays

Plasma and hepatic levels of TC, TG, and TBA were measured using commercial enzymatic assay kits from Nanjing Jiancheng Bioengineering Institute (TC: A111-1-1; TG: A110-1-1; TBA: E003-2-1, Nanjing, China).

### 4.3. Hematoxylin and Eosin (H&E) Staining

Liver samples were fixed in 4% paraformaldehyde for 24 h, embedded in paraffin, and sectioned. Subsequently, the sections were stained with hematoxylin and eosin (H&E) for histological observation under a microscope.

### 4.4. Immunofluorescence Staining

Paraffin-embedded liver tissues were sectioned at 6 μm thickness for immunofluorescence staining. Briefly, the sections were subjected to antigen retrieval using EDTA antigen retrieval buffer (pH 9.0) and then blocked with 10% goat serum for 30 min at room temperature (RT). Subsequently, the sections were incubated with the MPO antibody (A22900, ABclonal, Wuhan, China) overnight at 4 °C. On the following day, the sections were incubated with a fluorescently labeled 594-conjugated Goat anti-Rabbit IgG (AS039, ABclonal, Wuhan, China) and 4′,6-diamidino-2-phenylindole (DAPI) (for nuclear staining) at RT for 1 h, followed by observation under a fluorescence microscope.

### 4.5. Apoptosis Staining

Paraffin-embedded liver tissues were sectioned at 6 μm thickness, and these sections were stained using a Terminal Deoxynucleotidyl Transferase dUTP Nick-End Labeling (TUNEL) staining kit (Cat. A112-03, Vazyme, Nanjing, China) for the detection of apoptotic cells.

### 4.6. Immunohistochemical Staining

Paraffin-embedded liver tissue sections (6 μm thick) were used for immunohistochemical staining. Briefly, sections were subjected to antigen retrieval by heating in EDTA buffer (pH 9.0) in a pressure cooker for 10 min. Sections were blocked with 10% normal goat serum for 30 min at RT to prevent non-specific binding. Sections were then incubated overnight at 4 °C with IL6 primary antibody (A21264, ABclonal, Wuhan, China) diluted 1:100 in antibody dilution buffer. On the following day, sections were washed three times with phosphate-buffered saline containing 0.1% Tween-20 (PBST) and incubated with HRP-conjugated goat anti-rabbit IgG secondary antibody (AS014, ABclonal, Wuhan, China) for 30 min at RT. After another three washes with PBST, sections were developed with DAB substrate (Cat: 36302ES01, Yeasen, Shanghai, China) for 10 min, counterstained with hematoxylin for 7 min, and finally mounted and visualized under a light microscope.

### 4.7. Detection and Analysis of Intestinal Bile Acids-LC-MS/MS Analysis

Concentrations of bile acids in liver tissues and fecal samples were analyzed using ultra-high-performance liquid chromatography coupled with tandem mass spectrometry (UHPLC-MS/MS). The analysis was performed on a system consisting of a 1290 Infinity II UHPLC (Agilent Technologies, Santa Clara, CA, USA) coupled to a 6495 triple quadrupole mass spectrometer (Agilent Technologies). Chromatographic separation was achieved using a Waters ACQUITY UPLC BEH C18 column (2.1 × 100 mm, 1.7 µm; Waters, Milford, MA, USA) maintained at 40 °C. The mobile phase consisted of (A) water containing 7.5 mM acetic acid and (B) methanol/acetonitrile (1:1, *v*/*v*) containing 7.5 mM acetic acid, at a flow rate of 0.2 mL/min. A gradient elution program was used. Mass spectrometry detection was performed in negative ion mode using multiple reaction monitoring (MRM). Quantification was performed using external calibration curves prepared for each analyte. (LC-MS/MS analyses were performed by Mr. Hou Jinjun’s group at the Shanghai Institute of Pharmaceutical Sciences, Chinese Academy of Sciences).

### 4.8. RNA Sequencing (RNA-Seq) and Bioinformatic Analysis

Total RNA was extracted from fresh mouse liver tissues using TRIzol^®^ Reagent (Thermo Fisher Scientific, Waltham, MA, USA) according to the manufacturer’s protocol. Then, RNA quality was determined by the 5300 Bioanalyzer (Agilent) and quantified using the ND-2000 (NanoDrop Technologies, Wilmington, DE, USA). Only high-quality RNA sample (OD260/280 = 1.8~2.2, OD260/230 ≥ 2.0, RIN ≥ 6.5, 28S:18S ≥ 1.0, >1 μg) was used to construct sequencing library. Sequencing libraries were constructed and sequenced on the Illumina NovaSeq 6000 platform by Shanghai Majorbio Bio-pharm Biotechnology Co., Ltd. (Shanghai, China), following the standard protocol of the Illumina Stranded mRNA Prep, Ligation Kit. The raw paired-end reads were trimmed and quality controlled by FASTQ with default parameters. Then, clean reads were separately aligned to the reference genome with orientation mode using HISAT2 software (Version 2.2.1). Aligned reads were quantified using RSEM, and expression levels were normalized and expressed as Transcripts Per Million (TPM). Differentially expressed genes (DEGs) between groups were identified using the DESeq2 package with criteria of |log2FC| > 1 and false discovery rate (FDR) < 0.05. Functional annotation and pathway enrichment analyses of DEGs were performed for Gene Ontology (GO) terms and Kyoto Encyclopedia of Genes and Genomes (KEGG) pathways using GOATOOLS and KEGG Orthology Based Annotation System, respectively, with a corrected *p*-value (p_ fdr) ≤ 0.05 considered statistically significant. RNA sequencing data are available under NCBI SRA accession PRJNA1380070.

### 4.9. RT-qPCR

Total RNA was extracted from liver tissues or cells using Trizol reagent (Takara Bio Inc., Shiga, Japan) according to the manufacturer’s instructions. RNA concentration and purity were determined spectrophotometrically. Complementary DNA (cDNA) was synthesized from 1 µg of total RNA using the PrimeScript RT Master Mix (Perfect Real Time) (Takara Bio). Quantitative PCR (qPCR) was performed using Hieff qPCR SYBR Green Master Mix (Yeasen Biotechnology (Shanghai) Co., Ltd., Shanghai, China) on a 7500 Fast Real-Time PCR System (Applied Biosystems, Thermo Fisher Scientific, Waltham, MA, USA). The relative mRNA expression levels were calculated using the 2^−ΔΔCt^ method, with β-actin mRNA serving as the endogenous control. The sequences of the primers used are listed in Table 1.

### 4.10. Analysis of Gut Microbial Diversity

Fecal samples were collected from four rat groups (*n* = 3 per group): WT-CD, AHR^−/−^-CD, WT-HFHFrHCD, and AHR^−/−^-HFHFrHCD. Microbial DNA was extracted using the PF Mag-Bind Stool DNA Kit (Omega Bio-tek, Norcross, GA, USA), quantified via NanoDrop ND-2000 and Quantus Fluorometer (Promega, Madison, WI, USA), and stored at –80 °C. The 16S rRNA V3–V4 region was amplified with primers 338F/806R and sequenced on an Illumina PE300 platform (Majorbio, Shanghai, China). Raw reads were processed using FASTQ and FLASH, denoised into ASVs via DADA2 in QIIME2 (v2020.2), and taxonomically classified against SILVA 138 using a Naïve Bayes classifier. Samples were rarefied to 14,156 sequences (average good’s coverage: 97.90%). PICRUSt2 was used to predict metagenomic functions. Alpha and beta diversity (Bray–Curtis, PCoA) were calculated in Mothur and Vegan, respectively; PERMANOVA tested group differences. LefSe identified differentially abundant taxa (LDA > 2, *p* < 0.05). db-RDA with forward selection (9999 permutations) and linear regression assessed associations between clinical parameters and microbial profiles, with the VIF analysis to control for multicollinearity. Co-occurrence networks were built using Spearman’s correlation (|*r*| > 0.6, *p* < 0.05). Sequencing data are available under NCBI SRA accession PRJNA1380033.

### 4.11. Statistical Analysis

Statistical analyses were performed using GraphPad Prism (Version 10.0, GraphPad Software Inc., La Jolla, CA, USA). Data are presented as mean ± SD. Group comparisons were performed using the following tests: an unpaired two-tailed Student’s *t*-test for normally distributed data with equal variances, Welch’s *t*-test for normally distributed data with unequal variances, and the Mann–Whitney U test for non-normally distributed data. A *p*-value < 0.05 was deemed statistically significant.

## Figures and Tables

**Table 1:** Primer sequence.

Gene		Sequence (5′ → 3′)
*Cyp1a2*	Forward	AAGAGCGAGGAGATGCTCAA
	Reverse	TGCCGATCTCTGCCAATCAC
*Abcg8*	Forward	GATGCTGGCTATCATAGGGAGC
	Reverse	TCTCTGCCTGTGATAACGTCGA
*Apob*	Forward	AGAGGATCCCTGAGCAGGCTTCCTCAGCAG
	Reverse	TTAAAGCTTCAATGATTCTATCAATAATCTG
*Oatp1b2*	Forward	ACTACAAGTCAGCGGCTTCA
	Reverse	GGGTTCATTTTGGCGATTCC
*Abcb11*	Forward	AGCCAAAAGCTGAAAAGGTTGT
	Reverse	CTGGGCCATTCCATGTAGCA
*Angptl3*	Forward	TGACACCCAATCAGGCACTC
	Reverse	AGTGAGTGATGCAAGGAAATCAC
*Angptl4*	Forward	TTCTCTACCTGGGACCAAGA
	Reverse	CTGTAGTGGATAGTAGCGGC
*Adcy1*	Forward	TCACCCAGCCCAAGACGGATC
	Reverse	TCAGTAGCCTCAGCCACGGATG
*Ugt2b*	Forward	ATTTTGTCGGGACTGGCTGG
	Reverse	TGGTGGGCCTTCCCAAAATC
*Ugt2a1*	Forward	CCCTTGCCCAGATTCCTCAG
	Reverse	CTCTGGTTTTGGGATGTCCAAG
*Ugt2b7*	Forward	GCCCATCCTTGCCAAACATT
	Reverse	GTGCAAAGTCTTCCATTTCCTTA
*Hmgcr*	Forward	CCTCCATTGAGATCCGGAGG
	Reverse	GATGCACCGGGTTATCGTGA
*Srebf2*	Forward	TGCCTCACTCTCTGGAAAGG
	Reverse	GTAGGCCGCTGACATTGAG
*Abca1*	Forward	GGGTGGCTTCGCCTACTTG
	Reverse	GACGCCCGTTTTCTTCTCAG
*Scarb1*	Forward	GCATTCGGAACAGTGCAACA
	Reverse	TCATGAATGGTGCCCACATC
*Abcg5*	Forward	CGCAGGAACCGCATTGAAA
	Reverse	TGTCGAAGTGGTGGAAGAGCT
*Ntcp*	Forward	CATCGTGATGACCACCTGCT
	Reverse	TGGACTTGAGGACGATCCCT
*Mrp2*	Forward	TGCCCATTATCCGTGCCTTT
	Reverse	GAACAAAGCCCACAACGTCC
*β-actin*	Forward	CAGCTGAGAGGGAAATCGTG
	Reverse	CGTTGCCAATAGTGATGACC

## Data Availability

The RNA-seq and 16S rRNA gene sequencing data were deposited in the NCBI Sequence Read Archive (SRA) under BioProject accession numbers PRJNA1380070 (RNA-seq) and PRJNA138003 (16S rRNA). Temporary reviewer-access links are available at: RNA-seq: https://dataview.ncbi.nlm.nih.gov/object/PRJNA1380070?reviewer=9k21hv3fc616kgatg5q5nhmh9u (accessed on 12 December 2025). 16S rRNA: https://dataview.ncbi.nlm.nih.gov/object/PRJNA1380033?reviewer=64okm4u7ab66iaoiiqoi7re4bf (accessed on 12 December 2025).

## Supplementary Materials

The following supporting information can be downloaded at: https://www.mdpi.com/article/10.3390/ijms27010349/s1.

## Abbreviations

The following abbreviations are used in this manuscript:

AHR	Aryl hydrocarbon receptor
CA	Cholic acid
CD	Control diet
CDCA	Chenodeoxycholic Acid
DCA	Deoxycholic Acid
DEGs	Differentially expressed genes
GCA	Glycocholic Acid
GCDCA	Glycochenodeoxycholic Acid
GDCA	Glycodeoxycholic Acid
GHDCA	Glycohyodeoxycholic Acid
GLCA	Glycolithocholic Acid
GUDCA	Glycoursodeoxycholic Acid
HDLC	High-density lipoprotein cholesterol
HFHFrHCD	High-fat, high-fructose, high-cholesterol diet
IR	Insulin resistance
LCA	Lithocholic Acid
LDLC	Low-density lipoprotein cholesterol
MAFLD	Metabolic dysfunction-associated fatty liver disease
MPO	Myeloperoxidase
SD	Sprague-Dawley
TBA	Total bile acid
TC	Total cholesterol
TCA	Taurocholic Acid
TCDCA	Taurochenodeoxycholic Acid
TDCA	Taurodeoxycholic Acid
TG	Triglyceride
THCA	Taurohyodeoxycholic acid
TLCA	Taurolithocholic Acid
TUDCA	Tauroursodeoxycholic acid
UDCA	Ursodeoxycholic Acid
WT	Wild-type
