# Supplementary material for: AHR Deficiency Exacerbates Hepatic Cholesterol Accumulation via Inhibiting Bile Acid Synthesis in MAFLD Rats
Source: Int J Mol Sci. 2025 Dec 29;27(1):349. doi: 10.3390/ijms27010349 (PMC12785682; doi:10.3390/ijms27010349)
Supplement: Supplementary file 1 [file ijms-27-00349-s001.zip › ijms-3989332-supplementary.pdf]

AHR Deficiency Exacerbates Hepatic Cholesterol Accumulation via Inhibiting Bile Acid Synthesis in MAFLD Rats

Junjiu Xu [1,2,†], Pengwei Liu [3,4,†], Yuling Wu [1,4], Hongxiu He [1,2], Dandan Hu [4], Jianhua Sun[2,4], Jing Chen[2], Ying Tian [4,*], Likun Gong [1,2,3,4,5,*]

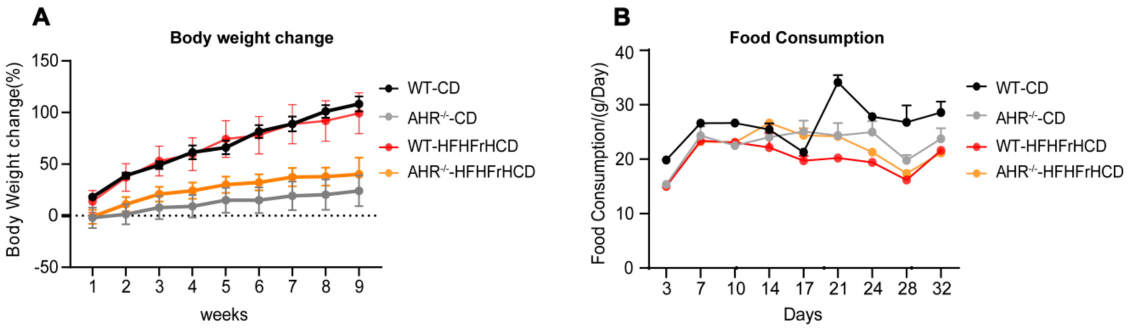

Figure S1. Weekly body weight change and food intake measurements by experimental group. (A) Body weight change (%). (B) Food intake.

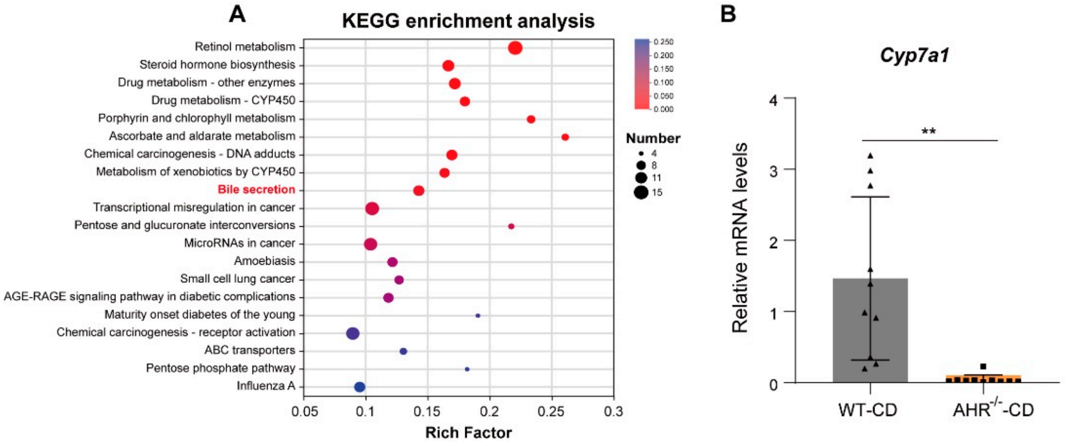

Figure S2. KEGG enrichment analysis and expression of associated genes in WT-CD versus AHR$^{-/-}$-CD groups. (A) KEGG enrichment analysis of DEGs in liver tissues between the WT-CD and AHR$^{-/-}$-CD groups. (B) Hepatic mRNA levels of Cyp7a1 in WT and AHR$^{-/-}$ rats fed a CD.

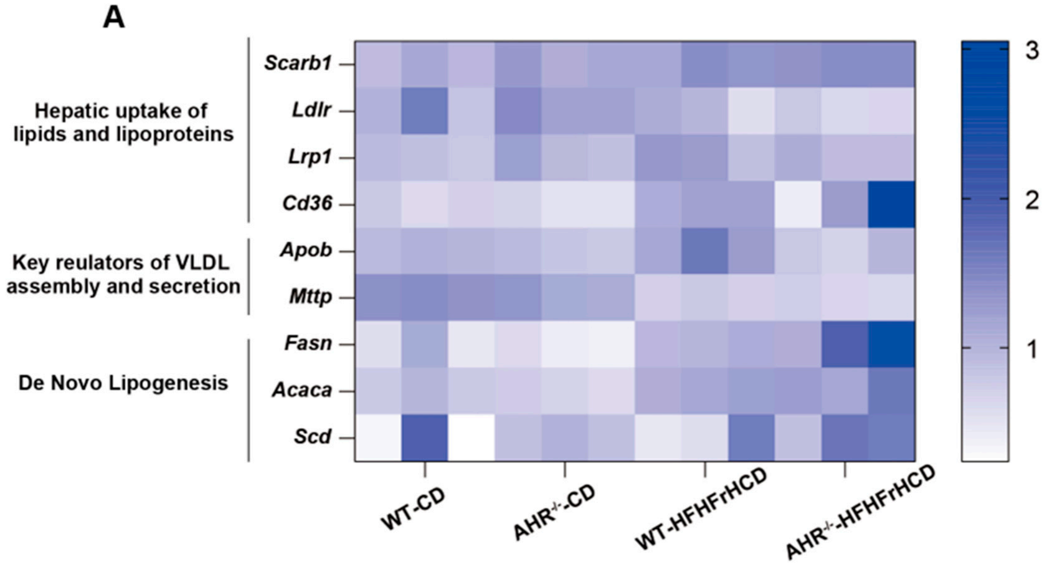

Figure S3. Hepatic gene expression heatmap of lipid metabolism–related genes.

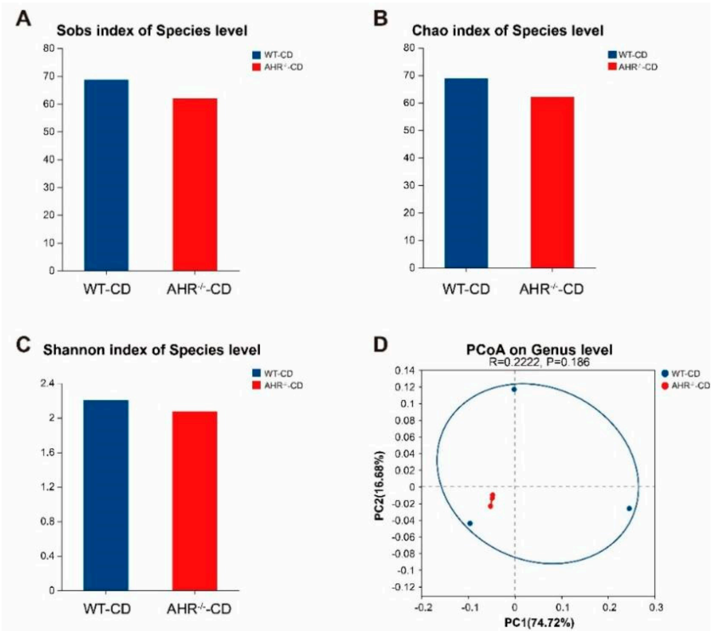

Figure S4. AHR knockout does not significantly alter gut microbiota composition in wild-type rats under standard housing conditions. (A-C) Alpha diversity index of gut microbiota at the species level in the WT-CD and AHR$^{-/-}$-CD groups: (A) Sobs, (B) Chao and (C) Shannon index. (D) PCoA analysis of gut microbiota at the genus level in the WT-CD and AHR$^{-/-}$-CD groups.
